# An approach to assess data-less small-scale fisheries: examples from Congo rivers

**DOI:** 10.1007/s11160-023-09770-x

**Published:** 2023-03-21

**Authors:** Leandro Castello, Felipe Carvalho, Nelly Ornelle Onana Ateba, Alidor Kankonda Busanga, Amy Ickowitz, Emmanuel Frimpong

**Affiliations:** 1 Department of Fish and Wildlife Conservation, Virginia Polytechnic Institute and State University, Blacksburg, VA USA; 2 Department of Parasitology and Ecology, University of Yaoundé 1, Yaoundé, Cameroon; 3 Department of Hydrobiology, University of Kisangani, Kisangani, Democratic Republic of the Congo; 4 Center for International Forestry Research, Bogor, Indonesia

**Keywords:** Inland fisheries, Life history, Local knowledge, Multispecies, Tropics, Shifting baselines

## Abstract

Small-scale fisheries (SSF) account for much of the global fish catch, but data to assess them often do not exist, impeding assessments of their historical dynamics and status. Here, we propose an approach to assess 'data-less' SSF using local knowledge to produce data, life history theory to describe their historical multispecies dynamics, and length-based reference points to evaluate stock status. We demonstrate use of this approach in three data-less SSFs of the Congo Basin. Fishers' recalls of past fishing events indicated fish catch declined by 65–80% over the last half-century. Declines in and depletion of many historically important species reduced the diversity of exploited species, making the species composition of the catch more homogenous in recent years. Length-at-catch of 11 of the 12 most important species were below their respective lengths-at-maturity and optimal lengths (obtained from Fishbase) in recent years, indicating overfishing. The most overfished species were large-bodied and found in the Congo mainstem. These results show the approach can suitably assess data-less SSF. Fishers' knowledge produced data at a fraction of the cost and effort of collecting fisheries landings data. Historical and current data on fish catch, length-at-catch, and species diversity can inform management and restoration efforts to curb shifting baselines of these fisheries. Classification of stock status allows prioritizing management efforts. The approach is easy to apply and generates intuitive results, having potential to complement the toolkits of researchers and managers working in SSF and engage stakeholders in decision-making processes.

**Supplementary Information:**

The online version contains supplementary material available at 10.1007/s11160-023-09770-x.

## Introduction

Small-scale fisheries (SSF) account for much of the global fish catch and contribute to the food security and nutrition of millions of people around the world, particularly in developing nations (Pauly and Zeller 2016; Fluet-Chouinard et al. 2018; Arthur et al. 2022). But SSF are poorly assessed and poorly managed, because governments in developing nations lack the data, science, and administrative structures necessary for fisheries management (Worm and Branch 2012; Pita et al. 2019). Achieving sustainability in SSF requires approaches to assess and facilitate their management in the socio-ecological contexts of developing nations (Andrew et al. 2007; McClanahan et al. 2009; Pita et al. 2019).

Many approaches have been developed to assess “data-poor” fisheries, but they suffer from at least one of two major limitations. Most such approaches require ‘some’ data. This is clearly illustrated in an edition of *Fisheries Research* dedicated to data-poor fisheries assessment approaches, where Jardim et al. (2015) defined a data-poor fishery as one lacking age-structured models capable of estimating a total allowable catch (Prince and Hordyk 2019). Such data requirements may be justifiable in developed nations but cannot be met in most SSF of the world (Andrew et al. 2007), many of which can be classified as "data-less", lacking any form of quantitative information (Johannes 1998). Most data-poor approaches also focus on providing 'snapshot' assessments that ignore historical trajectories, even though many SSF have dramatically affected the ecosystems and fish populations they exploit, particularly of large-bodied, slow-growing fishes (Pinnegar and Engelhard 2008). Ignoring historical degradation often leads to management efforts that perpetuate the shifting baseline syndrome, i.e., whereby each generation of fishers, managers, and scientists gets accustomed to progressively poorer fish assemblages (Pauly 1995; Soga and Gaston 2018). Data-less SSF require assessment approaches that, among other things, work in the absence of data and provide insights about their historical trajectories.

Here, we propose a general approach to assess the historical dynamics and status of data-less SSF. The approach builds on three well-established bodies of knowledge: local knowledge, life history theory, and length-based reference points. The novelty of the approach is in integrating these bodies of knowledge through a three-step process that produces data and assesses them for historical trends and fisheries status. Specifically, the approach produces historical data using surveys with fishers about their knowledge of past fish catches. It then assesses the historical dynamics of these tropical multispecies fisheries by analyzing the fishers' data based on insights from life history theory. Finally, the approach assesses the status of exploitation of key exploited species by comparing their length-at-catch against established length-based reference points of fishing performance.

Local knowledge is increasingly used to fill data gaps in fisheries (McElwee et al. 2020). A small but growing vein of this body of knowledge uses fishers' memories (i.e., recalls) of past fishing events to reconstruct timeseries data up to five decades in the past (e.g., Bender et al. 2014; Tesfamichael et al. 2014). Self-reported recalls of past events are susceptible to several biases due to imperfections of the human memory (Koriat et al. 2000). Many such biases relate to, or are part of, a range of issues that in recent environmental studies have been referred to as 'memory illusions' that distort recalled events (see Daw 2010). Overall, studies comparing fishers' recalls of fish catch to the range, or temporal trends, from monitoring landings data, found that they match general patterns (Gavin and Anderson 2005; Daw et al. 2011; Sáenz-Arroyo and Revollo-Fernández 2016). However, only about half of the studies that assessed fishers' recall and monitoring landings data for statistical relationships found support (Jones et al. 2008, 2020; Beegle et al. 2012; O’Donnell et al. 2012). The available evidence indicates fishers' recalls are not perfect substitutes for fisheries landing data but serve as useful proxies to identify general trends where no data exist (Castello 2023).

Life history theory predicts that fishing effects on fish assemblages vary across species. Biological traits such as large body size, late sexual maturity, and long generation time, among others, predict species' extinction vulnerability to fishing (Reynolds et al. 2005; Juan-Jordá et al. 2013; Mellin et al. 2016). Welcomme (1999) integrated insights from life history theory with the history of some SSF to propose the 'fishing down process', which differs from the fishing down marine food webs concept of Pauly et al. (1998), which focuses on trophic level. The fishing down process attempts to describe the historical dynamics of tropical multispecies fisheries, most of which are small-scale. It predicts that historical increases in effort leads to serial depletions of large-bodied, carnivore species (e.g., K-strategists), which are substituted by small-bodied species (e.g., r-strategists), which tend to be more productive (Welcomme 1999). This progressive "fishing down" lead to reorganization of exploited fish assemblages via declines in the catch of target species, mean size of target species, and diversity of exploited species (Lae 1997; Welcomme 1999; Lorenzen et al. 2006).

Length-based reference points can assess the exploitation status of SSF. Length-at-maturity has underpinned the notion that sustainable fishing requires allowing fish to grow to spawning size before harvesting (Prince and Hordyk 2019). Building on earlier work, however, recent studies show that fishing above length-at-maturity leads to sustainable fisheries not so much because it protects reproduction, but mostly because length-at-maturity nearly coincides with optimal length, the size where the interaction between body growth and natural mortality maximizes cohort biomass (Froese 2004; Cope and Punt 2009; Holt 2014). Optimal length nearly coincides with length-at-maturity, because energy used for growth in juveniles is mostly used for reproduction in adults (Holt 1958). Length-at-maturity and optimal length can be estimated from life history variables (Froese and Binohlan 2000) or obtained from FishBase (Froese and Pauly 2022) to assess if fishing adversely affects fish growth (i.e., recruitment-overfishing) or growth potential (i.e., growth-overfishing).

We demonstrate use of this approach in three data-less SSF in two rivers of the Congo Basin. First, we produced data by surveying fishers about their knowledge of past fish catch (multispecies and most caught species), length-at-catch of the most caught species, and species composition of the catch. We then assessed the historical dynamics of the fisheries by analyzing the local knowledge data for temporal changes in catch and diversity of exploited species. Finally, we assessed the status of exploited species by comparing their lengths-at-catch in recent years against lengths-at-maturity and optimal lengths.

## Methods

### Study fisheries

**Fig. 1:**
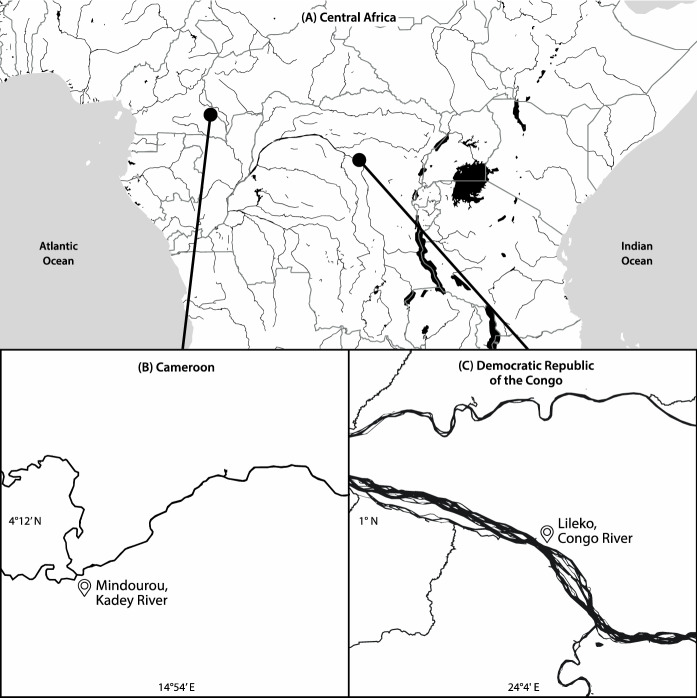
Location of the studied fisheries in **A** Congo Basin in Central Africa in **B** the Kadey River (Cameroon) and **C** the mainstem of the Congo River (Democratic Republic of the Congo)

We studied fisheries in Cameroon in the Kadey River, a tributary of the Congo Basin, and in the Democratic Republic of Congo on the mainstem Congo River (Fig. 1). These comprise data-less SSF for which, to our knowledge, no scientific data existed prior to our study. Fisheries in both sites relied on several types of gear, including gillnets, traps, hooks, and others; fishing was usually done by one fisher in the Kadey River and two fishers in the Congo River using pirogue canoes to fish for a few hours for sale or consumption, as is typical of SSF (Chuenpagdee and Pauly 2008). At the time of our fieldwork, fishing constituted an important source of food and livelihood for local people. In the Kadey site, fish were a key component of peoples’ diets and income, while it was the main animal source food in the Congo site.

The ecosystem in both sites is river-floodplain. In the Kadey River site, we sampled the Mindourou communities in the Ndélelé District. This is within the Sangha freshwater ecoregion (Thieme et al. 2005) in a forest-savanna transition zone, with a 100 m wide river channel and floodplain areas 5–20 m wide dominated by mixed forest and agricultural land. In the Congo River mainstem site, our study focused on the village of Lileko, near the mouth of the Lomami tributary River (near the city of Isangi). Lileko is situated within the large Cuvette Centrale freshwater ecoregion (Thieme et al. 2005), about 100 km from Kisangani, the capital of the Tshopo Province.

### Step 1: producing data

Our field team included three people in Cameroon and two people in the Democratic Republic of the Congo; the team surveyed fishers in each site for about one month, between February and August of 2021. The team approached fishers at landing sites or their homes, explained the nature of the research, and asked for their consent to ask questions about their knowledge of past fishing events. Interviews were done only after consent was given. Social distancing and Covid-19 mitigation procedures were followed. To avoid variability in the data across gear, the surveys focused on fishing done with gillnets, the most important gear in both rivers. The surveys considered (aggregate) multispecies catch, as well as catch of the five most caught species, which often contribute about two-thirds of the catch in weight in tropical SSF (Castello et al. 2011; Hallwass and Silvano 2016).

We developed our surveys using methods from other studies documenting fishers’ recall of fish catch (Sáenz-Arroyo et al. 2005; Jones et al. 2008, 2020; Daw et al. 2011; O’Donnell et al. 2012; Bender et al. 2014; Tesfamichael et al. 2014; Sáenz-Arroyo and Revollo-Fernández 2016; Thurstan et al. 2016; Early-Capistrán et al. 2020). There was one difference between our methods and those of some prior studies, which elicited recalls of 'good' and 'poor' fish catch, i.e., fish catches that are better or worse, respectively, than 'typical' catch, to minimize recall bias. The idea behind eliciting good and poor fish catch is focusing on more unique or memorable events that are thought to be less biased than typical fish catch (Tesfamichael et al. 2014). However, comparative analyses of these recall measures have produced varied results (Daw et al. 2011; O’Donnell et al. 2012; Thurstan et al. 2016). Given current uncertainty on the accuracy of different measures of recall of fish catch, our surveys elicited recalls of typical fish catch, which is more informative of past conditions than unique events. Fisheries are generally described by their prevailing patterns.

Our surveys produced data to describe historical changes in fish catch and diversity of exploited species. First, we asked fishers about the years when they started and stopped fishing with gillnets (if they still fished, 2020 was considered). Then, for the first and last three-year periods they fished, we asked fishers to describe their typical fishing trips in terms of catch (kg) and effort (measured in time spent fishing (hours)), for both multispecies (aggregate) catch and each of the five most caught species. Fishers reported common species names, and then identified the respective Linnaean taxonomic names using an image-based guide we showed to them (Table 1). Effort excluded travel time to and from fishing sites. For the five most caught species, we also asked fishers to rank their order of contribution in weight to total multispecies catch and estimate their average length-at-catch (cm) for the first and last three-year periods they fished. Finally, if fishers had been fishing with gillnets for more than 10 years, we also asked these same questions for a mid-point year in their careers. To account for possible effects induced by changes in gillnet length and mesh size, we also asked fishers to estimate gillnet length (m) and mesh size (cm, between opposing knots). The survey form used is shown in the Supplementary Information.

**Table 1:** The most important fishes targeted in each fishery and respective life-history traits and length-at-catch

River, fishery	Scientific name	Common name	Growth (K)	Natural mortality (M)	Age-at-maturity (year)	Maximum length (cm)	Length-at-catch (cm)	Ratio of length-at-catch to length-at-maturity
Kadey	*Distichodus mossambicus*	Mbengou	–	–	–	57	30	0.92^O^
	*Distichodus* sp.		–	–	–	–	25	–
	*Hydrocynus vittatus*	Ngòki	0.34	–	1.9	105	45	0.69^O^
	*Schilbe mystus*	Kembo	0.21	0.45	3.3	35	17.5	0.68^O^
	*Brycinus macrolepidotus*	Longò	0.46^a^	0.98	1.6	53	17	0.45^SO^
Congo, Small mesh	*Clarias gariepinus*	Ngolu	0.09	0.2	6.5	170	35	0.41^SO^
	*Brycinus grandisquamis*	Bakombo	0.49	–	1.5	26	6	0.37^SO^
	*Citharinus congicus*	Ndombolo	0.52	0.9	1.3	43	17.5	0.69^O^
	*Phenacogrammus interruptus*	Tutuko	1.87	3.55	0.5	8	5	1.02^H^
	*Heterotis niloticus*	Lamer	0.19	0.37	3.2	100	30	0.53^SO^
Congo, Large mesh	*Heterotis niloticus*	Lamer	0.19	0.37	3.2	100	30	0.53^SO^
	*Citharinus congicus*	Ndombolo	0.52	0.9	1.3	43	10	0.39^SO^
	*Distichodus lusosso*	Ekese	–	0.79	–	38	18	0.79^O^
	*Hydrocynus vittatus*	Mukobe	0.34	–	1.9	105	15	0.23^SO^
	*Distichodus antonii*	Mboto	–	0.64	–	55	30	0.95^O^

^a^Tah et al. (2010)

Except where indicated (^a^), parameters stem from the life-history tool of Fishbase (Froese and Pauly 2022). Common names are in Kako in the Kadey River and Lingala in the Congo mainstem. Length-at-catch data are medians for last three years. Species with a ratio of length-at-catch to length-at-maturity above 1 were here tentatively classified as ‘healthy’ (indicated with ^H^), those between 0.95 and 0.68 were classified as 'overexploited' (indicated with ^O^), and those with same ratio < 0.53 were classified as 'seriously overexploited' (indicated with ^SO^)

### Step 2: assessing historical dynamics

We assessed the historical dynamics of the fisheries by analyzing the local knowledge data with respect to temporal changes in catch and diversity of exploited species. To assess if catch declined over time, we fitted linear mixed regression models (LMM) that had catch as the response variable and year, gillnet length, and time spent fishing as candidate explanatory variables. The models included each interviewed fisher as a random effect to allow the intercept to vary randomly by fisher. Catch was log-transformed while candidate explanatory variables, which were all continuous, were standardized (using the function scale of the R software; R Core Team 2020). Year was estimated as the mid-point of the first and last three-year periods fishers fished, as well as the mid-point between the first and last years they fished. The models included gillnet length and fishing time as covariates because they can influence catch. The decision to fit linear models came from observing that log-transformed catch followed linear trends over time. Although linear trends cannot describe future trends in catch, since catch cannot be negative, here we were only concerned with describing past trends. We fitted the models using the restricted maximum likelihood estimation (REML) method in R (Team 2013), with the nlme package (Pinheiro et al. 2007). We computed all possible models, including catch in all models and allowing for interactions involving only catch. We then averaged the top models with a Δ AIC < 4 (using the *dredge()* function of the MuMIn package; Bartoń 2016). For species that had an effect of year on catch, we plotted predicted LMMs excluding effects of gillnet length and time spent fishing. Predicted catches were plotted on back-transformed scale for more intuitive interpretation of results.

We assessed if model assumptions were met using four diagnostics tests: To assess the assumption of linearity, we evaluated residuals vs. fitted values plots. To assess the assumption of normality, we evaluated QQ-plots of theoretical vs. sample quantiles. To assess the assumption of independence, we evaluated for potential autocorrelation (particularly as our historical catch reconstructions could be susceptible to temporal correlations) using lagged plots of residuals vs. lagged residuals. Finally, to assess the assumption of lack of correlation among predictor variables, we computed Variance Inflation Factor for each fitted coefficient. Diagnostic tests showed model assumptions were met; see Supplementary information.

 Finally, to assess if the diversity of exploited species declined over time, we pursued a data-driven characterization of the species composition of each fishery. First, we determined if there were clusters of similar species and relative abundances in the catch based on the top 12 most harvested species reported by all fishers in each fishery. In these analyses, we focused on a larger number of species than in the above catch and length analyses to avoid inadvertently constraining the raw data and producing misleading results. Then, we related the identified clusters to year of harvest to assess the effect of time as a factor in the clustering; if there was evidence of the effect of time, we estimated the median year when a different species composition occurred. Finally, we calculated cluster averages of species abundances to describe the catch composition for each period associated with each cluster.

We implemented this approach by conducting a k-means cluster analysis using the R package *cluster*. The k-means clustering algorithm allows the user to specify a priori the number of clusters in the species relative abundance data. The entities being grouped are grouped iteratively to achieve a configuration that minimizes within-group variation and maximizes between-group variation for the specified number of clusters. We varied the a priori specified number of clusters from 1 to 10 and determined the best number of clusters by several criteria that assess how well within-cluster variation is minimized and between-cluster variation is maximized. For each dataset, we calculated the Manhattan distance matrix among all survey responses on species composition to form the clusters and determined the optimal number of clusters using three criteria (within sum of squares, silhouette, and gap statistics), which were built into the *cluster* package. Once we determined the optimal number of clusters, we used analysis of variance (ANOVA) to test for effect of time on the clustering, followed by post hoc analyses using Tukey’s honestly significant difference tests. We then compared species composition between clusters using multivariate analysis of variance (MANOVA).

### Step 3: assessing fisheries status

In the final step of our assessment approach, we assessed the status of the fisheries by comparing length-at-catch of the five most caught species for the last three years against length-at-maturity and optimal length. Because studies on growth and reproduction for most species in our fisheries are rare, we obtained length-at-maturity from the Life-History Tool of FishBase (Froese and Pauly 2022) and estimated optimal length based on Froese and Binohlan (2000), unless noted.

## Results

### Fishers' knowledge data

We interviewed 329 fishers (115 in the Kadey River and 214 in the Congo mainstem), producing 893 observations of multispecies catch, 4360 observations of catch and length-at-catch for each of the five most caught species, and 4360 observations of species' ranks in the catch. For one taxa (*Distichodus* sp*.*; Table 1) in the Kadey River, we could identify only the genus but not the species name.

### Historical dynamics

#### Kadey River

**Fig. 2:**
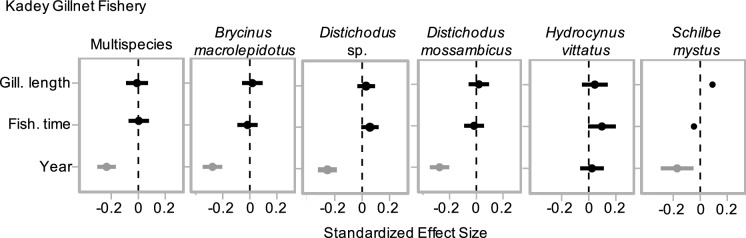
Effect of year, fishing time (hr), and gillnet length (m) on catch (kg) of the five most caught species in the Kadey River in Cameroon. Shown are parameter estimates of the slope of each explanatory variable and respective confidence intervals (95%). Relationships with slopes that do not overlap 0 are colored grey

**Fig. 3:**
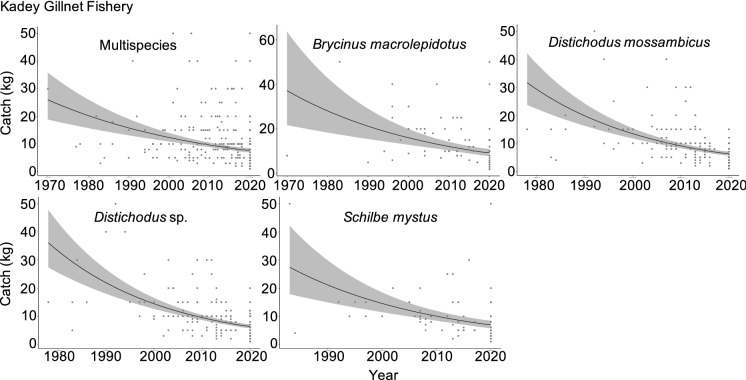
Historical declines in catch in the Kadey River. Total catch, where (black) line is predicted linear mixed model (LMM), overlaid with points showing 'raw' catch estimates. 95% confidence interval shown in grey. Only species that had an effect of year on catch are shown. Catch is plotted on back-transformed scale for more intuitive interpretation

Our assessment of the historical dynamics of the fishery in the Kadey River revealed declining trends in recalled catch. The five most caught species were: *Brycinus macrolepidotus*, *Distichodus sp.*, *Distichodus mossambicus*, *Hydrocinus vittatus* and *Schilbe mystus*. Over a 51-year period, multispecies catch declined by 65%, from ~ 26 kg in 1970 to ~ 9 kg in 2019 (Figs. 2, 3). The catch of four (*Brycinus macrolepidotus*, *Distichodus sp.* and *Distichodus mossambicus* and *Schilbe mystus*) of the five most caught species also declined, while the catch of *Hydrocinus vittatus* remained stable (Figs. 2, 3).

**Fig. 4:**
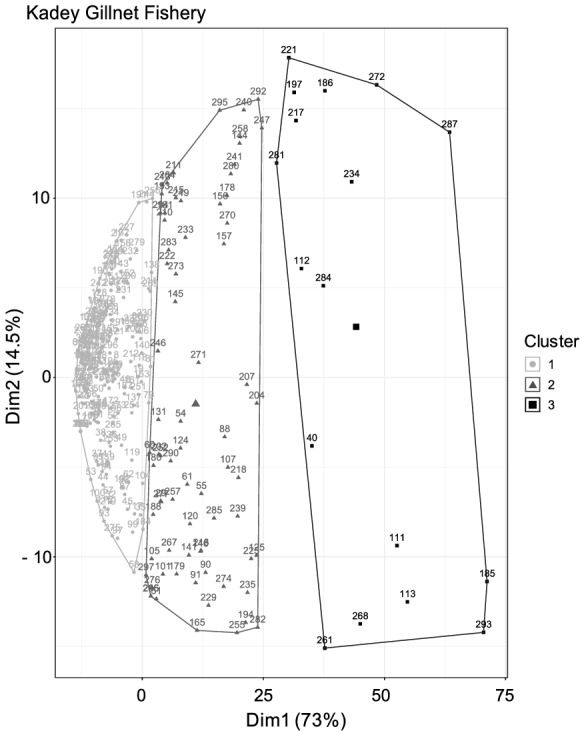
Clusters of species composition of the catch in the Kadey River in Cameroon. The three species clusters had a funnel pattern pointing to the left, indicating a reduction in species diversity over time

**Fig. 5:**
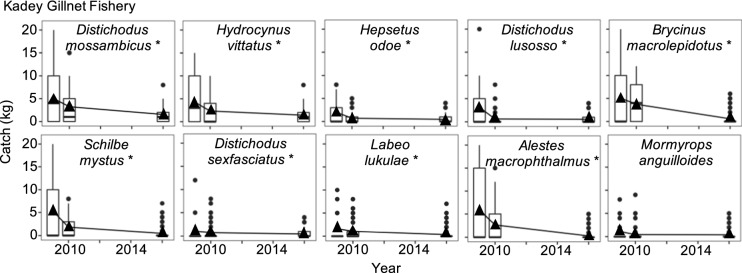
Catch of the 10 most caught species in the Kadey River during three time periods of distinct species composition, as determined by cluster analysis. Asterisks indicate change in catch over time according to comparisons of species abundance and composition by cluster using MANOVA (*p* < 0.05). Filled triangles indicate the average catch and filled circles indicate outliers. Data reveal declines of many species that once dominated fish catches

Fish catch in the Kadey River also suffered declines in species diversity. We found three clusters of species in the catch (Fig. 4), which were correlated with year (ANOVA, df = 294, *p* < 0.05; see Fig. 1S). These species clusters had a funnel pattern, indicating the species composition became more homogenous over time, with no species dominating the catch in recent years (Fig. 4). This homogenization was driven by declines of nine (*Distichodus mossambicus*, *Distichodus sp.*, *Hydrocinus vittatus*, *Distichodus lusosso*, *Brycinus macrolepidotus*, *Schilbe mystus*, *Distichodus sexfasciatus*, *Labeo lukulae*, and *Alestes macrophthalmus*) of the 10 most caught species between 1970 and 2019, as indicated by MANOVA (*p* < 0.001; Fig. 5).

#### Congo mainstem

There were two gillnet fisheries with distinct mesh sizes in the Congo mainstem. The gillnet fishery with a small mesh (mean = 2.1 cm) existed from 1959 until 2019, targeting *Brycinus grandisquamis*, *Citharinus congicus*, *Clarias gariepinus*, *Heterotis niloticus*, and *Phenacogrammus interruptus*. In 1980, fishers also began operating a gillnet fishery with a large mesh (mean = 7.7 cm), targeting *Distichodus antonii*, *Distichodus lusosso*, *Hydrocinus vittatus*, *Citharinus congicus* and *Heterotis niloticus*.

**Fig. 6:**
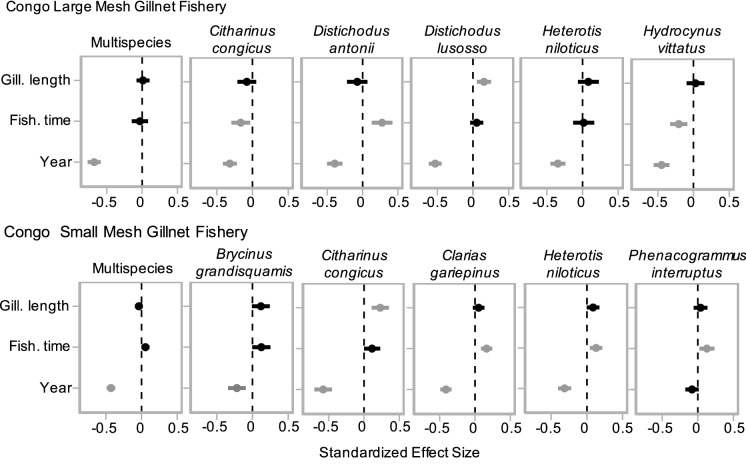
Effect of year, fishing time (hr), and gillnet length (m) on catch (kg) of the five most caught species in the large and small mesh gillnet fisheries in the Congo mainstem. Shown are parameter estimates of the slope of each explanatory variable and respective confidence intervals (95%). Relationships with slopes that do not overlap 0 are colored grey

**Fig. 7:**
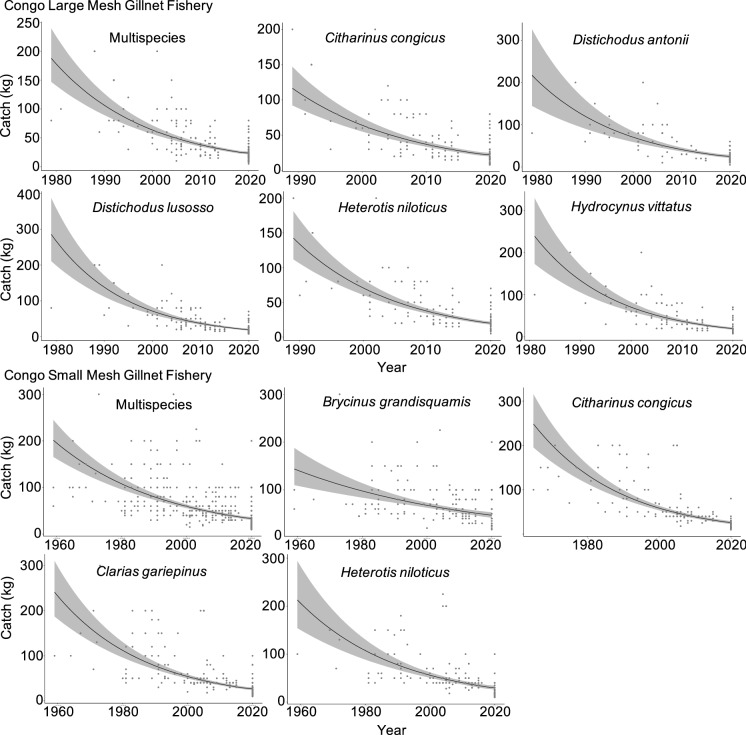
Historical declines in catch in the large and small mesh gillnet fisheries of the Congo mainstem. Total catch, where (black) line is predicted linear mixed model (LMM), overlaid with points showing 'raw' catch estimates. 95% confidence interval shown in grey. Only species that had an effect of year on catch are shown. Catch is plotted on back-transformed scale for more intuitive interpretation

Fish catch has declined dramatically in the Congo mainstem in both types of gillnet fisheries. The multispecies catch of the large mesh gillnet fishery declined by 84% over a 39-year period, from ~ 190 kg in 1980 to ~ 30 kg in 2019 per fishing time (Figs. 6, 7). In the small mesh gillnet fishery, multispecies catch declined by 80%, from 200 kg in 1959 to 40 kg in 2019 (Figs. 6, 7). These catch declines were substantially greater in magnitude than in the Kadey River, and they were observed in seven of the eight most caught species in both fisheries (Fig. 6, 7).

**Fig. 8:**
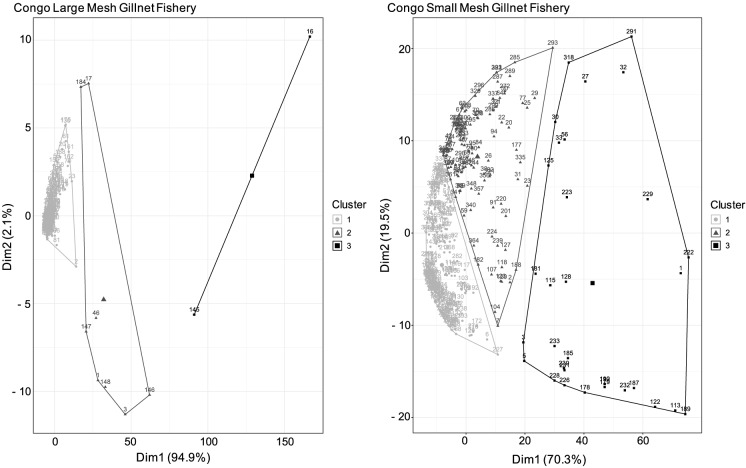
Clusters of species composition of the large (left panel) and small (right panel) mesh fisheries in the Congo mainstem. The three species clusters had a funnel pattern pointing to the left, following a progression over time toward decreased diversity of species

**Fig. 9:**
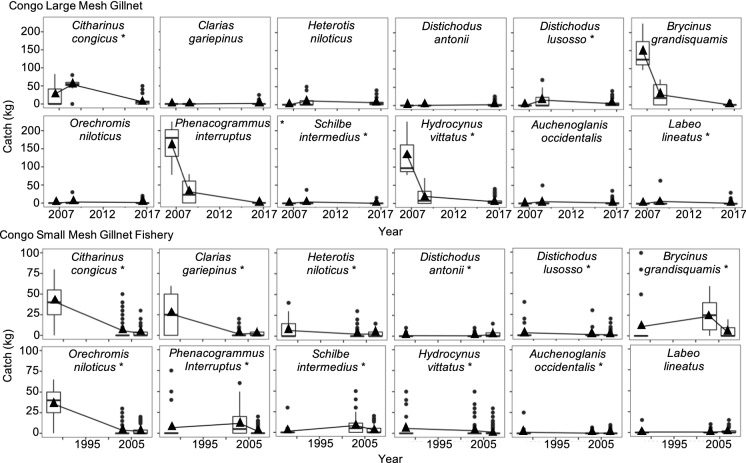
Catch of the 12 most caught species from the large and small mesh gillnet fisheries in the Congo mainstem during three periods of distinct species composition, as determined by cluster analysis. Asterisks indicate change in catch over time according to comparisons of species abundance and composition by cluster using MANOVA (*p* < 0.05). Filled triangles indicate the average catch and filled circles indicate outliers. The general pattern reveals declines of species that once dominated fish catches

Fish catch in both fisheries in the Congo mainstem suffered reductions in species diversity. We found three clusters of species in both fisheries. The clusters were correlated with year for small mesh gillnet fishery (ANOVA, df = 365, *p* = 0.001), but they were not correlated with year for the large mesh gillnet fishery (ANOVA, df = 226, *p* = 0.46). This implies a temporal trend in the small mesh gillnet fishery but not in the large mesh gillnet fishery (Fig. 2S). However, species clusters for both fisheries exhibited funnel patterns (Fig. 8), indicating and suggesting in the small and large mesh gillnet fisheries, respectively, that species composition became more homogenous over time in both fisheries, with no particular species dominating the catch in recent years. This homogenization in the large mesh gillnet fishery was driven by declines in abundance of seven (*Citharinus congicus*, *Distichodus lusosso*, *Brycinus grandisquamis*, *Phenacogrammus interruptus*, *Schilbe intermedius*, *Hydrocynus vittatus* and *Labeo lineatus*) of the 12 most caught species, as indicated by MANOVA (*p* < 0.05; Fig. 9). In the small mesh gillnet fishery, this homogenization was driven by declines (large and small) in abundance of 11 (*Citharinus congicus*, *Clarias gariepinus, Heterotis niloticus*, *Distichodus antonii*, *Distichodus lusosso*, *Oreochromis niloticus*, *Brycinus grandisquamis*, *Phenacogrammus interruptus*, *Schilbe intermedius*, *Hydrocynus vittatus* and *Auchenoglanis occidentalis*) of the 12 most caught species, as indicated by MANOVA (*p* < 0.001; Fig. 9).

### Fisheries status

**Fig. 10:**
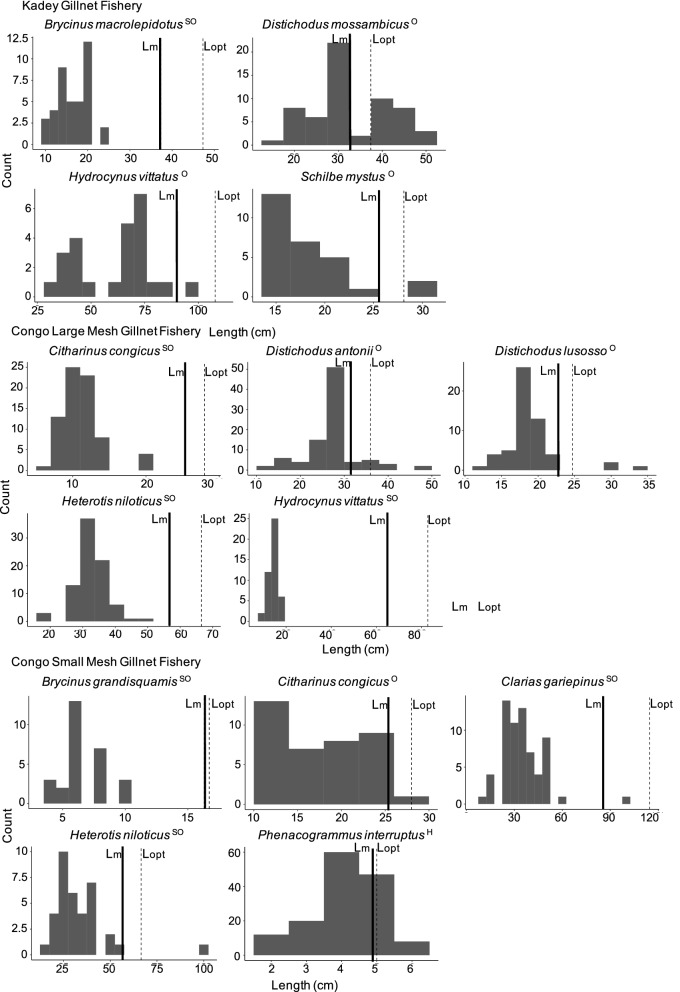
Assessment of stock status based on two length-based reference points: length-at-maturity (Lm; indicated with continuous line) and optimal length (Lopt; indicated with dashed line). Histograms compare length-at-catch of most caught species in each fishery against Lm and Lopt. As shown in other studies (Froese 2004; Prince and Hordyk 2019), median length-at-catch (shown in Table 1) equal or greater than Lopt denote a 'healthy' (^H^) stock; if it is below Lopt or Lm, it is 'overfished' (^O^). In our study, many species were fished at half or less their respective Lm or Lopt (see details in Table 1), suggesting the stocks were 'seriously overfished' (^SO^). These classifications of stock status (^H^, ^O^, or ^SO^) are indicated next to each species name

Most of the most important species were overfished. In the Kadey River, all four species we could identify taxonomically (*Brycinus macrolepidotus, Schilbe mystus*, *Hydrocynus vittatus* and *Distichodus mossambicus*) were caught below their length-at-maturity and optimal length in recent years (Fig. 10; Table 1). In both fisheries of the Congo mainstem, seven of the eight most caught species (*Distichodus mossambicus*, *Clarias gariepinus*, *Brycinus grandisquamis*, *Citharinus congicus*, *Heterotis niloticus*, *Distichodus lusosso*, *Hydrocynus vittatus* and *Distichodus antonii*) were caught below their lengths-at-maturity and optimal lengths in recent years; only one species (*Phenacogrammus interruptus*; Fig. 10) was caught at its length-at-maturity and optimal length (Fig. 10; Table 1).

Some species were more overfished than others, as indicated by differences between length-at-catch and length-at-maturity. Of all species in the three fisheries, seven could be classified as overfished as their lengths-at-catch were on average 82%, or just below, their lengths-at-maturity. In contrast, seven species could be classified as seriously overfished as their lengths-at-catch were on average only 41%, or substantially below, their lengths-at-maturity (Table 1). Geographically, this pattern manifested with six out of eight species in the Congo mainstem being seriously overfished, compared with one out of four in the Kadey River. Biologically, this pattern manifested as a function of species body sizes. Average maximum length of seriously overfished species was twice as large (81.8 cm, 100 median) as that of overfished species (40 cm, 43 median; Table 1).

## Discussion

### Fisheries sustainability in the Congo Basin

Our results indicate that fisheries in two rivers of the Congo Basin have undergone key transformations during the last half century, following unsustainable trajectories. Fishers' recall data indicate fish catch declined markedly, both at the multispecies and individual species levels. Those catch declines and depletion of many historically important species induced reductions in the diversity of target species, with the species composition of the catch tending to be more homogenous and no species dominating the catch in recent years. These trends resulted in 11 of the 12 most important species being overfished. Although there was some variation, species responses to fishing were mediated by their body sizes, with large-bodied species being the most overfished. Without management interventions, these fisheries will likely continue to decline, requiring increasing fishing effort and becoming increasingly dependent on a reduced number of small fishes.

These trends are worrying given that Cameroon and the Democratic Republic of the Congo suffer from substantial food insecurity and malnutrition (Global Nutrition Report 2021) in large part due to poor diets. Fish are affordable animal source foods that are rich in micronutrients, essential amino acids, and fatty acids (Thilsted et al. 2016; Hicks et al. 2019). Potential reductions in fish consumption would likely exacerbate current rates of malnutrition.

Preventing further degradation of these fisheries requires management action. Legislation that establish bans on fishing gears and closed seasons or moratoria for some species exist in both countries.^1^ However, most existing regulations focus on coastal and marine environments or have not been implemented in our study sites. If implementation deficiencies could be addressed, gear, size, and season regulations could promote sustainability. Protected areas, no-take reserves, and fish reserves could also contribute to making fisheries more sustainable (Koning et al. 2020). One strategy to foster sustainability could be following the rule of thumb of regulating size selectivity of the gillnets so that fish catch concentrates on specimens at optimal length (Prince and Hordyk 2019). This strategy is increasingly recognized as an ideal management strategy because it maintains catch and spawning biomass at high levels even with intense fishing effort (Cope and Punt 2009; Froese et al. 2016; Prince and Hordyk 2019). This strategy could be expensive to fishers if it requires replacing their gillnets, but it is easier to enforce compared with size, season, or area regulations (McClanahan and Mangi 2004).

### Advantages and limitations of the approach

We proposed and demonstrated the use of an approach to assess the historical dynamics and status of data-less SSF. The approach addresses key limitations of other data-poor fisheries assessments, by dispensing with the need for prior data and providing a historical perspective. We believe the approach can adequately assess data-less SSF, although with some limitations.

*Producing data:* Fishers' knowledge produced data quickly and cost-effectively. One month of survey work by five people produced thousands of observations of catch, length-at-catch, and species' ranks in the catch at a fraction of the cost of collecting fisheries landings data. It also produced data for the past half century, which is impossible in most situations except via the use of historical evidence (McClenachan et al. 2012; Pauly and Zeller 2016).

Using fishers' recalls of past fishing events to produce fisheries data has two limitations. First, it is often difficult to identify taxa based on common names (e.g., *Distichodus* sp*.* in the Kadey River). This limitation is not unique to fishers' knowledge, as taxonomic inventories are poor in the tropics, requiring care. Second, fishers' recalls of past fish catch can be biased. One type of bias is induced by human motives, as fishers can elicit higher- or lower-than-real catches to qualify for subsidies or to follow a community narrative (e.g., an oil spill 10 years ago ruined our fishery; Papworth et al. 2009). Such biases are difficult to account for and can impede production of reliable data. In the fisheries studied herein, we are unaware of motives for fishers to bias their recalls. The other bias type stems from cognitive processes of the human memory of past everyday events (i.e., episodic memory; Koriat et al. 2000), which likely affect all recalls of fish catch. These biases remain poorly understood (Diamond et al. 2020). However, the view emerging from prior studies is that fishers' recalls of past fish catches are informative proxies that describe general patterns, not perfect substitutes for fisheries landing data. That fishers' recalls can describe historical patterns is seen in the tight confidence intervals of our models of historical fish catch, which indicate consistency in the data. Did multispecies catch in Congo fisheries decline by exactly 65–80% as our models indicate? Until further evidence on the accuracy of fishers' recalls becomes available, we would caution about interpreting these results literally and believe that it is safer to infer that catch in the fisheries studied here declined several-fold. This uncertainty can be uncomfortable to some, but we note that monitoring of landings data can be equally (un)reliable. Official data by the Food and Agriculture Organization of the United Nations underestimates global fish catch by 53% and report biased historical and spatial trends at multiple spatial scales, even in the data-rich regions (Pauly and Zeller 2016).

Despite these limitations, it seems obvious to us that using fishers' recalls data affected only by the biases of the human memory is far more desirable than the alternative of "no data". For decades, the global dearth of fisheries data has led to repeated calls for more data and science (e.g., Ovando et al. 2021), even as developing nations have been unable to produce it. Belief in the dominant view that only data-rich scientific assessments can guide decision-making has precluded use of alternative sources of information, preventing much-needed policy action.

*Describing historical patterns:* Our proposed analysis of fishers' knowledge data, guided by insights from life history theory, can produce valuable information about historical dynamics of multispecies fisheries. Unlike "snapshot" assessments that ignore historical trajectories, the approach can inform on the magnitude, rate, and extent of historical changes on fish assemblages induced by fishing. A snapshot assessment would likely have missed the documented declines in catch and diversity of targeted species. Fisheries conservation efforts that do not consider such historical changes could inadvertently perpetuate fish assemblages and associated catches to poor conditions. The historical information produced by our approach can serve as targets for restoration and management efforts (Humphries and Winemiller 2009) to curb shifting baselines of fisheries in the Congo Basin, at least as far back as our data go. This is important because fishing effort in small-scale fisheries has been growing globally (Rousseau et al. 2019), and many small-scale fisheries have induced large ecosystem impacts (Pinnergard and Engelhard 2008). Use of short catch timeseries datasets induces biases in determination of reference points and classification of stock status (Schijns and Pauly 2022).

### Outlook for application

Our approach is relatively easy to apply, mostly requiring familiarity with surveys of fishers' knowledge, analysis of historical trends, and length-based reference points. As shown for SSF in the Congo Basin, the approach can produce useful descriptions of historical dynamics and classification of the exploitation status of key taxa. While results have limitations, the approach probably delivers the bulk of the outcome at a fraction of the effort of conventional assessments. The approach, therefore, can complement the toolkits of researchers and managers working in SSF, particularly when there is interest in their historical dynamics but there are no data.

A bonus of this approach is its potential to foster engagement in management by local stakeholders. Unlike most stock assessment approaches, our simple approach––based on fishers' knowledge, historical trends, and size comparisons––can likely be understood by stakeholders with no formal training. The use of data that come from the knowledge of the fishers themselves can also be expected to improve decision-making and fishers' participation in management. As observed in cases where decisions rely on fishers' knowledge (e.g., Castello et al. 2009), this is because fishers tend to accept management decisions made based on their own knowledge more than those based on scientific analyses they do not comprehend. If fishers have a clear understanding of how their fisheries "got there" in the past, they are better positioned to decide where their fisheries "need to go" in the future. Engaging fishers in the research process is a first step towards their involvement in better managing their fisheries. This is even more critical when government agencies are largely absent.

## Supplementary Information

Below is the link to the electronic supplementary material.

Supplementary file1 (DOCX 1216 KB)

## Data Availability

The datasets generated during and analyzed in this study are available from the corresponding author on reasonable request.

## Footnotes

1 In Cameroon, legislation includes Decree No. 95/413/PM of June 20, 1995; Order No. 0002/MINEPIA of August 1, 2001, in Article 6 of Chapter III; and law n°94/01 of January 20, 1994 on Forestry, Wildlife and Fisheries Regime. In the Democratic Republic of the Congo, legislation includes articles 61, and 62, and 65 of the Colonial Decree of April 21, 1937; and Ordinance of January 9, 1981.
