# Supplementary material for: An approach to assess data-less small-scale fisheries: examples from Congo rivers
Source: Rev Fish Biol Fish. 2023 Mar 21:1–18. Online ahead of print. doi: 10.1007/s11160-023-09770-x (PMC10030197; doi:10.1007/s11160-023-09770-x)
Supplement: Supplementary file 1 — Supplementary file1 (DOCX 1216 KB) [file 11160_2023_9770_MOESM1_ESM.docx]

**Supplementary Information**

An approach to assess data-less small-scale fisheries: examples from Congo rivers

Authors

Leandro Castello^1,^ *^,∅^, Felipe Carvalho^1,∅^, Onana Ateba Nelly Ornelle^2^, Alidor Kankonda Busanga^3^, Amy Ickowitz^4^, Emmanuel Frimpong^1^

^1^ Department of Fish & Wildlife Conservation, Virginia Polytechnic Institute and State University, Blacksburg, Virginia, United States

^2^ Department of Parasitology and Ecology, University of Yaoundé 1, Yaoundé, Cameroon

^3^ Department of Hydrobiology, University of Kisangani, Kisangani, Democratic Republic of the Congo

^4^ Center for International Forestry Research, Bogor, Indonesia

* Corresponding author: E-mail: leandro@vt.edu

^∅^ These authors contributed equally to this work

**Field survey**

Survey used to interview fishers about historical trends in catch, length-at-catch, and species composition of the catch. Survey shown in English but applied in French (and sometimes in Kako (Kadey River) or Lingala (Congo River) depending on the fisher.

| For how many years have you been fishing with gillnets? _______________ |
| --- |

In last 3 years fishing

| What was the average catch (kg) for all species together for each time the gillnets were checked? ___________________  How many hours (hr) on average were the gillnets in the water before checking them? ___________________  What was the average length of the gillnet? ___________________(m)  What was the average mesh size (between of opposing knots) of the gillnet?__________ (cm)  What were the 5 most important species harvested (in total weight)?  What was the average catch (kg) of top 5 species for each time the gillnets were checked?  And what was their average length (cm)?   \|  \| Name (in decreasing order) \| Catch (kg) \| Length (cm) \| \| --- \| --- \| --- \| --- \| \| sp 1 \|  \|  \|  \| \| sp 2 \|  \|  \|  \| \| sp 3 \|  \|  \|  \| \| sp 4 \|  \|  \|  \| \| sp 5 \|  \|  \|  \| |
| --- | --- | --- | --- | --- | --- | --- | --- | --- | --- | --- | --- | --- | --- | --- | --- | --- | --- | --- | --- | --- | --- | --- | --- | --- |

First three years fishing; approximate year:___________

| What was the average catch (kg) for all species together for each time the gillnets were checked? ___________________  How many hours (hr) on average were the gillnets in the water before checking them? __________________  What was the average length of the gillnet? ___________________(m)  What was the average mesh size (between of opposing knots) of the gillnet?__________ (cm)  What were the 5 most important species harvested (in total weight)?  What was the average catch (kg) of top 5 species for each time the gillnets were checked?  And what was their average length (cm)?   \|  \| Name (in decreasing order) \| Catch (kg) \| Length (cm) \| \| --- \| --- \| --- \| --- \| \| sp 1 \|  \|  \|  \| \| sp 2 \|  \|  \|  \| \| sp 3 \|  \|  \|  \| \| sp 4 \|  \|  \|  \| \| sp 5 \|  \|  \|  \| |
| --- | --- | --- | --- | --- | --- | --- | --- | --- | --- | --- | --- | --- | --- | --- | --- | --- | --- | --- | --- | --- | --- | --- | --- | --- |

IF fisher has fished for more than 10 years; then years in "middle" of fishing career: ________

| What was the average catch (kg) for all species together for each time the gillnets were checked? ___________________  How many hours (hr) on average were the gillnets in the water before checking them? ___________________  What was the average length of the gillnet? ___________________(m)  What was the average mesh size (between of opposing knots) of the gillnet?__________ (cm)  What were the 5 most important species harvested (in total weight)?  What was the average catch (kg) of top 5 species for each time the gillnets were checked?  And what was their average length (cm)?   \|  \| Name (in decreasing order) \| Catch (kg) \| Length (cm) \| \| --- \| --- \| --- \| --- \| \| sp 1 \|  \|  \|  \| \| sp 2 \|  \|  \|  \| \| sp 3 \|  \|  \|  \| \| sp 4 \|  \|  \|  \| \| sp 5 \|  \|  \|  \| |
| --- | --- | --- | --- | --- | --- | --- | --- | --- | --- | --- | --- | --- | --- | --- | --- | --- | --- | --- | --- | --- | --- | --- | --- | --- |


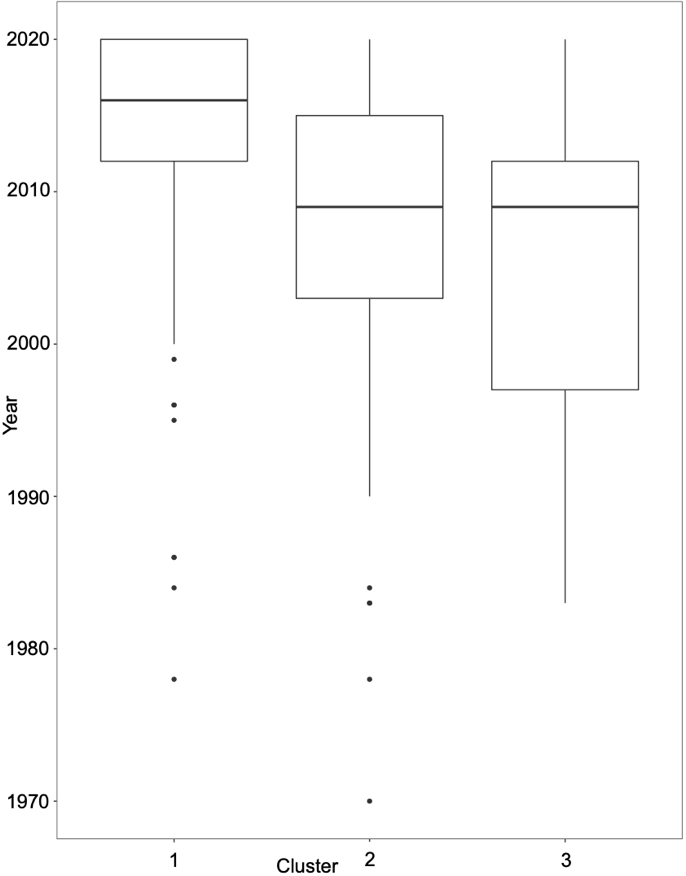


**Figure S1** Relationship between the clusters of species composition from the Kadey River in Cameroon (Fig. 4) with their respective years. The Tukey HSD test indicated that clusters 2 and 3 had a different year from cluster 1 (p < 0.05), but the two clusters were not significantly different from each other (p > 0.05).


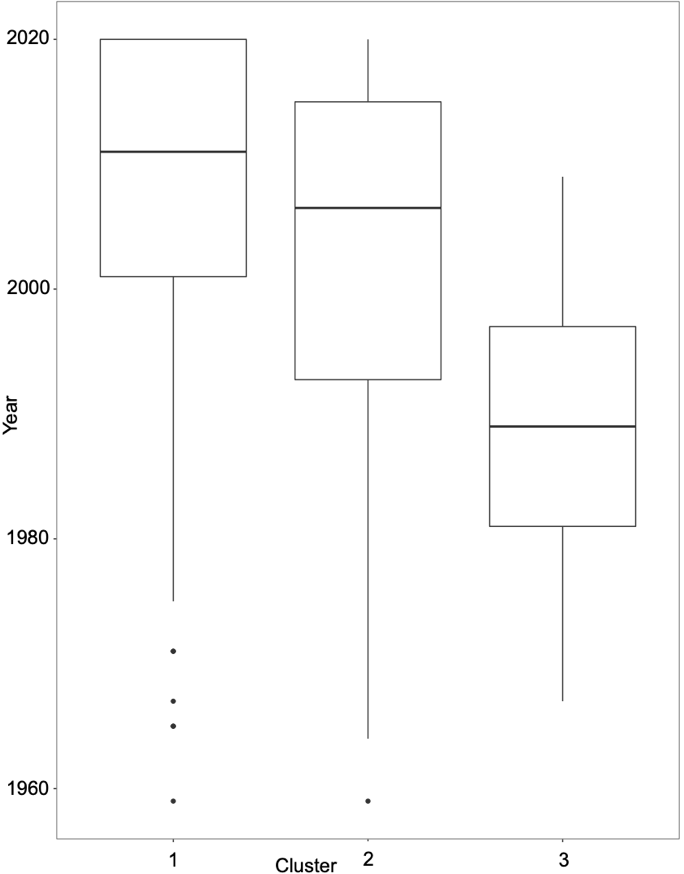

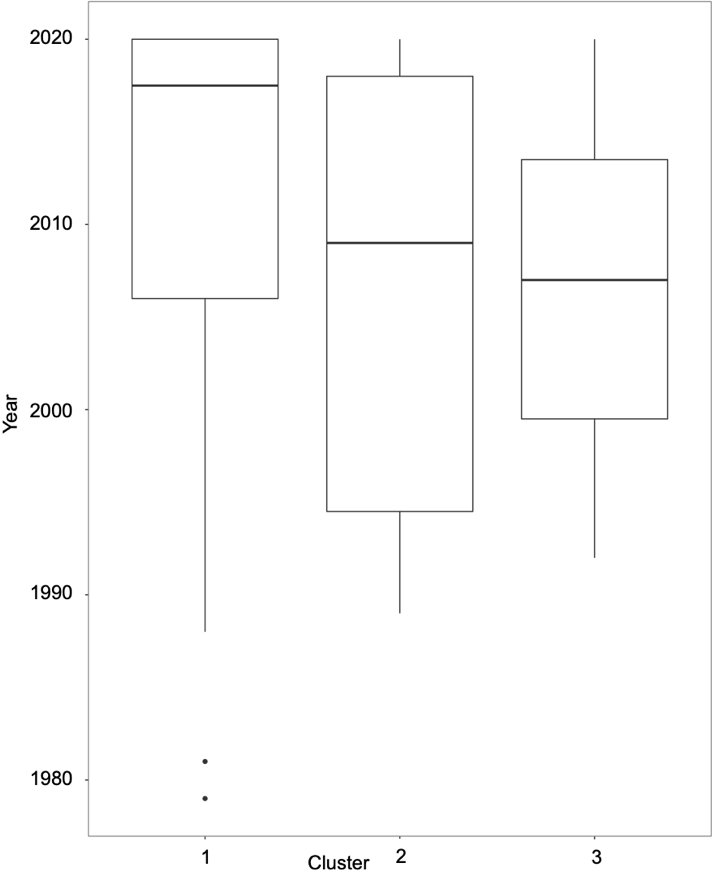


**Figure S2** Relationship between the clusters of species composition from the Congo River in Cameroon (Fig. 8) with their respective years. Left panel is for the small mesh gillnet fishery and right panel is for the large mesh gillnet fishery. In the small mesh gillnet fishery, the Tukey HSD test indicated that clusters 1 and 2 had different year from cluster 3 (p < 0.001), but the two clusters were not significantly different from each other (p > 0.05). In the large mesh gillnet fishery, clusters had weak evidence of temporal differences (p = 0.06).

**Regression diagnostics**

**Table S1** Variance inflation factors (VIF) for each predictor variables in our regression models of catch. VIF values start at 1; values greater than 5 indicate multicollinearity between predictor variables

| River, Fishery | Model | Year | Fishing time | Gillnet length |
| --- | --- | --- | --- | --- |
| Kadey River, gillnet fishery | Multispecies | 1.008 | 1.021 | 1.029 |
|  | *Distichodus mossambicus* | 1.010 | 1.012 | 1.002 |
|  | *Distichodus* sp. | 1.010 | 1.029 | 1.019 |
|  | *Brycinus macrolepidotus* | 1.027 | 1.059 | 1.087 |
|  | *Schilbe mystus* | 1.033 | 1.030 | 1.019 |
|  | *Hepsetus odoe* | 1.060 | 1.065 | 1.022 |
| Congo River, small mesh gillnet fishery | Multispecies | 1.327 | 1.184 | 1.172 |
|  | *Brycinus grandisquamus* | 1.397 | 1.489 | 1.500 |
|  | *Citharinus congicus* | 1.163 | 1.029 | 1.154 |
|  | *Clarias gariepinus* | 1.263 | 1.175 | 1.284 |
|  | *Heterotis niloticus* | 1.152 | 1.070 | 1.097 |
|  | *Phenacogrammus interruptus* | 1.296 | 1.628 | 1.399 |
| Congo River, large mesh gillnet fishery | Multispecies | 1.235 | 1.788 | 1.667 |
|  | *Citharinus congicus* | 1.350 | 1.598 | 1.699 |
|  | *Distichodus antonii* | 1.283 | 2.334 | 2.237 |
|  | *Distichodus lusosso* | 1.444 | 1.159 | 1.531 |
|  | *Heterotis niloticus* | 1.275 | 2.027 | 2.090 |
|  | *Hydrocynus vittatus* | 1.169 | 1.168 | 1.349 |

**Regression diagnostic plots**

Kadey River gillnet fishery

Multispecies

*Distichodus mossambicus*

*Distichodus* sp.

*Brycinus macrolepidotus*

*Schilbe mystus*

*Hydrocynus vittatus*

Congo River, large mesh gillnet fishery

Multispecies

*Citharinus congicus*

*Distichodus antonii*

*Distichodus lusosso*

*Heterotis niloticus*

*Hydrocynus vittatus*

Congo River small mesh gillnet fishery

Multispecies

*Brycinus grandisquamus*

**

*Citharinus congicus*

*Clarias gariepinus*

*Heterotis niloticus*

*Phenacogrammus interruptus*
